# Potential vectors of equine arboviruses in the UK

**DOI:** 10.1136/vr.103825

**Published:** 2016-09-30

**Authors:** G. E. Chapman, D. Archer, S. Torr, T. Solomon, M. Baylis

**Affiliations:** 1 Epidemiology and Population Health, Institute of Global Health, University of Liverpool, Liverpool, UK; 2 Vector Biology, Liverpool School of Tropical Medicine, Liverpool, UK; 3 Clinical Infection, Microbiology and Immunology, Institute of Global Health, University of Liverpool, Liverpool, UK

**Keywords:** Brain diseases, Horses, Neurology, Notifiable diseases, Virology

## Abstract

There is growing concern about the increasing risk of disease outbreaks caused by arthropod-borne viruses (arboviruses) in both human beings and animals. There are several mosquito-borne viral diseases that cause varying levels of morbidity and mortality in horses and that can have substantial welfare and economic ramifications. While none has been recorded in the UK, vector species for some of these viruses are present, suggesting that UK equines may be at risk. The authors undertook, therefore, the first study of mosquito species on equine premises in the UK. Mosquito magnet traps and red-box traps were used to sample adults, and larvae were collected from water sources such as tyres, buckets, ditches and pools. Several species that are known to be capable of transmitting important equine infectious arboviruses were trapped. The most abundant, with a maximum catch of 173 in 72 hours, was *Ochlerotatus detritus*, a competent vector of some flaviviruses; the highest densities were found near saltmarsh habitats*.* The most widespread species, recorded at >75 per cent of sites, was *Culiseta annulata.* This study demonstrates that potential mosquito vectors of arboviruses, including those known to be capable of infecting horses, are present and may be abundant on equine premises in the UK.

Globally, there is increasing concern over emerging infectious diseases, particularly arthropod-borne viruses (arboviruses) affecting human beings and livestock (Kilpatrick and Randolph 2012, Durand and others 2013). Examples from the UK include bluetongue and Schmallenberg viruses in ruminants. The introduction of West Nile virus (WNV) into North America demonstrated the effects of mosquito-borne disease on a naive host population, both human and equine, and concerns have also been raised over the potential for spread of other mosquito-borne arboviruses affecting horses (Brown and others 2008, Pages and others 2009, Durand and others 2013). Mosquito-borne arboviruses affecting horses include WNV, Japanese encephalitis virus (JEV), Eastern equine encephalitis virus (EEEV), Western equine encephalitis virus (WEEV), Venezuelan equine encephalitis virus (VEEV), Ross River virus (RRV), Murray Valley encephalitis virus (MVEV) and Getah virus (Table 1).

**TABLE 1:** Mosquito-borne viruses affecting horses and known morbidity and mortality information

	Virus							
	JEV	WNV	EEEV	WEEV	VEEV	MVEV	RRV	Getah virus
Inapparent infections common	Yes^9^	Yes^7^	Yes^2^	Yes	No^9^	Yes^13^	Yes^12^	Yes
Morbidity	0.03–1.4% of horses in a region^3^	1 in 11–12 infections^7^	61%^1^ of horses on some farms	low	10% of regional population (estimated)^10,11^	Low	Low	Unknown
Case mortality	5–40%^4,5^^,6^	38–57%^7^	Up to 73%^1^	20–30%^8^	40–90%^10,11^	Low	Low	Not fatal
Vaccination available	Y	UK licensed	Y	Y	Y			Y

Y—available in affected countries

^1^Silva and others (2011)

^2^Pauvolid-Corrêa and others (2010)

^3^Spickler (2010)

^4^Ellis and others (2000)

^5^Hale and Witherington (1953)

^6^Nakamura (1972)

^7^Sellon and Long (2013)

^8^Long and Gibbs (2007)

^9^Rico-Hesse (2000)

^10^Sudia and others (1975)

^11^Zehmer and others (1974)

^12^Vale and others (1991)

^13^Holmes and others (2012)

EEEV, Eastern equine encephalitis virus; JEV, Japanese encephalitis virus; MVEV, Murray Valley encephalitis virus; RRV, Ross River virus; VEEV, Venezuelan equine encephalitis virus; WEEV, Western equine encephalitis virus; WNV, West Nile virus

Further knowledge about potential vector mosquitoes in the UK and their ability to spread arboviruses will play a key role in control and surveillance of disease in the event of an outbreak. Climate change may increase the risk of emergence of arboviral diseases by several mechanisms. Higher temperatures increase the ability of vectors to transmit viruses (Guis and others 2012, MacKenzie-Impoinvil and others 2015) and also have the potential to increase the geographical range of mosquitoes (Elbers and others 2015). Increased winter rainfall may also contribute to increase in mosquito populations due to creation of more temporary freshwater sites for breeding and greater abundance of emerging mosquitoes in spring (Vardoulakis and Heaviside 2012).

There are 34 species of mosquito in the UK (Medlock and Vaux 2011) and species that are implicated as vectors of arboviruses of horses elsewhere in the world include *Aedes cinereus, Aedes vexans, Anopheles maculipennis s.l*., *Coquillettidia richiardii*, *Culex pipiens, Culex modestus*, *Culiseta morsitans, Ochlerotatus caspius, Ochlerotatus dorsalis and Ochlerotatus flavescens* and a number of these are widely distributed and locally abundant across the UK (Table 2)*.* In addition, some mosquito species present in the UK have been shown in the laboratory to be competent vectors of at least one of these viruses including *Ochlerotatus punctor, Oc. detritus, Cx. modestus, Ae. vexans, Cx. pipiens s.l.* and *Anopheles plumbeus* (Table 2).

**TABLE 2:** Mosquito species present in the UK, horse and mammal biting, and vector status for arboviruses of horses

Species	Host biting^5,7^	Evidence of equine biting	Vector status
*Aedes cinereus/Aedes geminus*	M^31, 32^ B^31, 32^	Morocco^1^, Switzerland^32^	EEEV (I)^18^
***Aedes vexans***	**M^31, 32^**,**B^31,32^**	**France^2^**,**Switzerland^32^**	**WNV (I)^5^**,EEV (I, L*)^18,19,20,21,22,^
*Anopheles algeriensis*	M		
***Anopheles claviger***	**M^32^**	**Switzerland^32^**	
***Anopheles maculipennis s.l.***	**M^31,32^**, **B^31^**	**UK^4,8^**, **Switzerland^32^**	**WNV (I)^5^**
***Anopheles plumbeus***	**M^31^**	**France^2^**	**WNV (L)^14^**
***Coquillettidia richiardii***	**M^31,32^**, **B^32^**	**France^2^, Switzerland^32^**	**WNV (I)^5^**
*Culiseta alaskaensis*	M		
***Culiseta annulata***	**M^14,32^**, **B^32^**	**UK^3^, France^2^**, **Switzerland^32^**	**WNV (L)^33^**
*Culiseta fumipennis*	B		
*Culiseta litorea*	M, B		
*Culiseta longiareolata*	B		
***Culiseta morsitans***	**M^31^**, **B^31^**		**EEV (Z)^17, 19^**
***Culiseta subochrea***	**M^2^**	**France^2^**	
*Culex europaeus*	A, R, B		
*Culex modestus*	M^2^, B^2^	France^2^	WNV (V, L)^2.5^
***Culex pipiens s.l.***	**M^31^ B^31,32^**	**France^2^**	**WNV (V, L)^23, 27^**, **JEV (L†)^23^**, **EEV (N)^26^**, **WEEV (N)^24,25^**, **VEEV (N)^27^**
***Culex torrentium***	**B^31^**, **M^31^**		
*Ochlerotatus annulipes*	M^9,31^, B	France^2^	
***Ochlerotatuscantans***	**M^31^**, **B^32^**	**UK^9^**, **Switzerland^32^**	
***Ochlerotatus caspius***	**M^2^**, **B^2^**	**UK^3^, France^2^**	**WNV (I, L†**)**^2,5^**
*Ochlerotatus communis*	M^31^		
***Ochlerotatus detritus***	**M^2^**, **B**	**UK^3^, France^2^**	**WNV (L)^16^**, **JEV (L)^13^**
***Ochlerotatus dorsalis***	**M^6^**	**UK^6^**	**WEEV (I, L)^28,30^**
*Ochlerotatus flavescens*	M^11,12^	Denmark, Canada^11, 12^	
*Ochlerotatus geniculatus*	M^2,31^	France^2^	
*Ochlerotatus leucomelas*			
***Ochlerotatus punctor***	**M^10^**, **B**	**UK^10^**	**WNV (L)^14^**
***Ochlerotatusrusticus***	**M^31, 32^**, **B**	**Switzerland^32^**	
*Ochlerotatus sticticus*	M^31, 32^, B^31^	Switzerland^32^	
*Orthopodomyia pulcripalpis*	B		

Species in bold were sampled during the present study

*Variable laboratory competence in a number of studies

†Relatively inefficient laboratory vector

^1^Faraj and others (2009)

^2^Balenghien and others (2006)

^3^Pilot work for this study—site 8, 2014

^4^Danabalan (2010)

^5^Medlock and others (2005)

^6^Service (1971a)

^7^Becker and others (2010)

^8^Hutchinson (2004)

^9^Medlock and Vaux (2011)

^10^Service and others (1986)

^11^Service and Smith (1972)

^12^Rempel and others (1946)

^13^MacKenzie-Impoinvil and others (2015)

^14^Vermeil and others (1960)

^15^Balenghien and others (2008)

^16^Blagrove and others, (2016)

^17^Andreadis and others (1998)

^18^Armstrong and Andreadis (2010)

^19^Centers for Disease Control and Prevention (CDC) (2006)

^20^Vaidyanathan and others (1997)

^21^Davis (1940)

^22^Chamberlain and others (1954)

^23^Turell and others (2006)

^24^Aviles and others (1990)

^25^Hammon and Reeves (1943)

^26^Merrill and others (1934)

^27^Turell (2012)

^28^Kramer and others (1998)

^29^Vaux and others (2015)

^30^Zacks and Paessler (2010)

^31^Börstler and others (2016)

^32^Schönenberger and others (2016)

^33^M.S.C. Blagrove, personal communication

A, amphibians; B, birds; EEEV, Eastern equine encephalitis virus; I, implicated in disease transmission worldwide; JEV, Japanese encephalitis virus; L, laboratory-competent vector; M, mammals; N, non-competent as laboratory vector; R, reptiles; V, ecologically significant bridge vector worldwide; VEEV, Venezuelan equine encephalitis virus; WEEV, Western equine encephalitis virus; WNV, West Nile virus; Z, ecologically significant enzootic vector worldwide

In the UK, there have been recent and ongoing sampling and surveillance of mosquito species (Snow and Medlock 2008, Medlock and Vaux 2013, 2014, 2015, Vaux and Medlock 2015, Vaux and others 2015); however, there has been no sampling of mosquito species with specific focus on the equine host. Accordingly, the authors carried out a survey of the mosquitoes present at 32 premises across England (see Fig 1 for approximate locations) to obtain baseline data on the species composition and abundance of mosquitoes that may interact readily with equines. The authors’ results identify which species may play an important role in outbreaks of mosquito-borne equine viruses in the UK and hence contribute to the development of national strategies to monitor and manage this risk.

**FIG 1:**
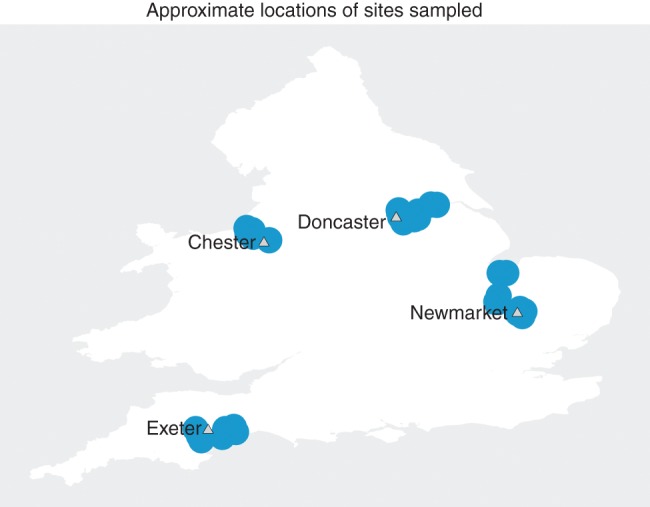
Map of study areas

## Methods

A total of 32 sites were sampled—8 equine premises in each of northwest, northeast, southeast and southwest regions in England (Fig 1). A stratified sampling approach was used due to the fact that many mosquito species have a patchy population distribution. In this case, four types of mosquito breeding habitat were identified: land associated with drainage ditches (drained farmland) or fenland (site 29), woodland, urban and salt marsh (Hutchinson and others 2007). The authors aimed to recruit two equine premises in each of the four habitats in each region (32 premises in total).

An internet search was conducted using Google Maps and The Phone Book from British Telecom, using the search terms ‘Riding Schools’, ‘Livery’, ‘Stables’, ‘Stud’. BHS Riding Schools and Livery Yard Lists and the British Equestrian Directory and Newmarket Trainers Association lists were also used**.** This produced a list of businesses with publicly available contact details.

For each premises, the local area was investigated for potential mosquito habitats using Magic (www.magic.gov.uk) and Google Earth. Sites were assigned a category based on habitat (some sites qualified for two categories) and were graded based on area of presumed habitat and proximity of habitat to the premises. The authors aimed to locate premises within suitable habitats or, if that was not possible, within 500 m (woodland and urban sites), 1 km (urban and drained farmland sites) or 3 km (salt marsh). A maximum distance of 500 m for woodland sites was selected reflecting the relative ease of finding sites close to woodland. For salt marsh or grazing marsh, it was not possible to find sites in close proximity in many cases, but species associated with floodwater, such as *Ae. vexans* and coastal salt marsh such as *Oc. detritus*, tend to have greater dispersal capacity and *Oc. detritus* is capable of flying at least 2.5 miles (Service 1969, 1971b, Snow and Medlock 2008, Becker and others 2010, Verdonschot and Besse-Lototskaya 2014). In order to try and include all four habitat types within reasonable travelling distance, the four areas within the regions were chosen as follows: Wirral peninsula and Chester (northwest); between Scunthorpe, Gainsborough, Doncaster and Goole (northeast); within 20 miles of Exeter (southwest); a transect from Newmarket to the Wash (southeast).

Premises were recruited by sending out either a letter or email to the business, and following up with a telephone call. For sites where there was no response or a negative response, correspondence was then sent to a number of alternative second-choice sites, for that category of habitat, until 32 sites (eight in each of four regions) were recruited.

### Mosquito sampling

#### Host-seeking adults

Each of the 32 sites was visited three times throughout the summer of 2015, and mosquitoes were trapped continuously for three days. Timing of visits was based on what is presently known about peaks in adult mosquito numbers of different species in the UK, visiting each of four regions within each of three seasonal peaks of mosquito activity in the months of May, late June–early July and September (Service 1969, 1977, Medlock and others 2007, Snow and Medlock 2008, Becker and others 2010, Medlock and Vaux 2015).

Trapping at each site was carried out using a mosquito magnet, Independence model (Woodstream Europe). The mosquito magnet is designed to catch host-seeking mosquitoes by using propane as a fuel source to produce heat, moisture and carbon dioxide. The trap was also baited with 1-octen-3-ol (as supplied by the trap manufacturer). The mosquito magnet trap was run continuously for ∼72 hours starting in the morning and a data logger was placed underneath the body of the trap to record the environmental temperature and relative humidity for this time period. The traps were emptied as time allowed, so that the samples were not in the trap for more than 48 hours, to reduce damage, and the subsamples were combined and identified as the full 72-hour catch.

Attempts were made to catch mosquitoes landing on hosts in order to confirm horse biting. These attempts were made in the afternoon and wherever possible at dusk (Service 1969, 1971a). To fit in with the trapping schedule, four sites in each area were sampled in June/July and September in the mid-late afternoon, and four sites around dusk. Therefore, one site per day was sampled in the afternoon and one in the evening for Monday to Thursday of the trapping week in each sampling area. For each sampling effort, a group of horses was observed for 15 minutes to see whether any mosquitoes could be identified landing on them. If no mosquitoes were observed, then another group of horses was observed for 15 minutes. Group sizes ranged from 1 to 10 as horses were in their normal grazing environment (with the exception of site 25 where sampling was attempted in the stable as there was no grazing). If no mosquitoes were observed on two groups, the attempt was abandoned. If mosquitoes were observed, landings were counted for 2 minutes and then mosquitoes sampled from the head and neck of the horse (for reasons of safety) for 30 minutes to allow for species identification. Some premises could not be sampled at dusk due to access restrictions, so were only sampled in the afternoon. In order to trap mosquitoes feeding on horses, a mechanical pooter (Watkins and Doncaster) was modified with an elongated inlet tube and was muffled so as to avoid startling the horse. Individual horse behaviour was discussed with the yard owner in advance, and permission to attempt landing catches with each horse or group of horses was obtained.

#### Resting adults

**FIG 2:**
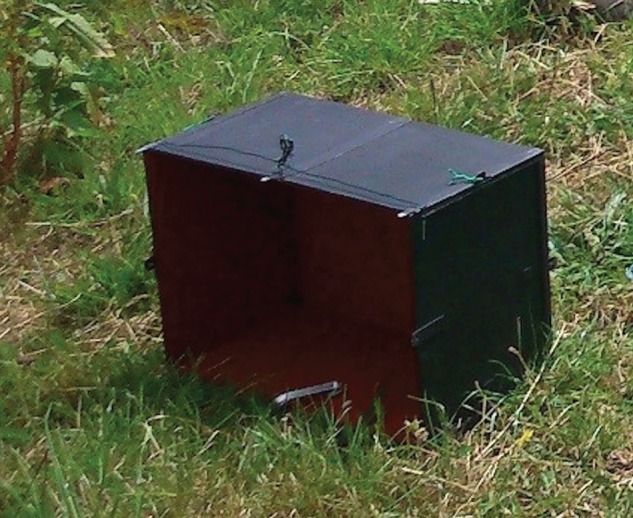
Red-box trap

The resting box trap was a 40×30×20 cm black box (Morris 1981), painted red inside (red-box trap) and was designed to aid in the capture of blood-fed mosquitoes (Fig 2). It was set in an open area facing west and was emptied on two mornings (either at 24 and 72 hours after deployment or 48 and 72 hours) by placing a perspex cover on the open front of the box and aspirating resting mosquitoes.

#### Immature mosquitoes

Larval sampling was undertaken on the equine premises themselves and, where there was access, on neighbouring land within 500 m of the mosquito magnet or of grazing horses. The aim was to sample all water sources within the boundary of the premises, including all collections of artificial containers. This was not always possible due to access constraints or on larger premises. Larvae and pupae were sampled using a dipper. This is a 500 ml container with a long handle that is used to sample water sources. Each dip was then emptied into a white tray and searched for larvae. For larger water bodies, 5×500 ml dips were used in different parts of the water body, whereas for small containers only one dip sample or partial dip samples could be obtained.

### Sample handling and identification

Mosquitoes were removed from the traps with a mechanical aspirator and ‘Fly-nap’ (Carolina Biological Supply Company) was used to produce knockdown. Adult mosquitoes were stored dry and identified within four days. Blood-fed mosquitoes were stored in 90 per cent ethanol immediately.

Larvae were pipetted into universal containers for storage. Fourth instar larvae were killed by gradually adding 90 per cent ethanol. Pupae were allowed to emerge for ease of identification. Live second and third instar larvae were allowed to continue to develop until the end of the fieldwork week for ease of identification. Containers were inspected daily and any dead larvae or pupae were preserved using 90 per cent ethanol for identification (Snow 1991).

Mosquitoes of all stages were identified morphologically as far as possible to species or species complex using keys of British and European mosquitoes (Marshall 1938, Cranston and others 1987, Snow 1991, Schaffner and others 2001, Becker and others 2010). *Cx. pipiens* was differentiated from *Culex torrentium* by molecular methods as described by Hesson and others (2010).

**FIG 3:**
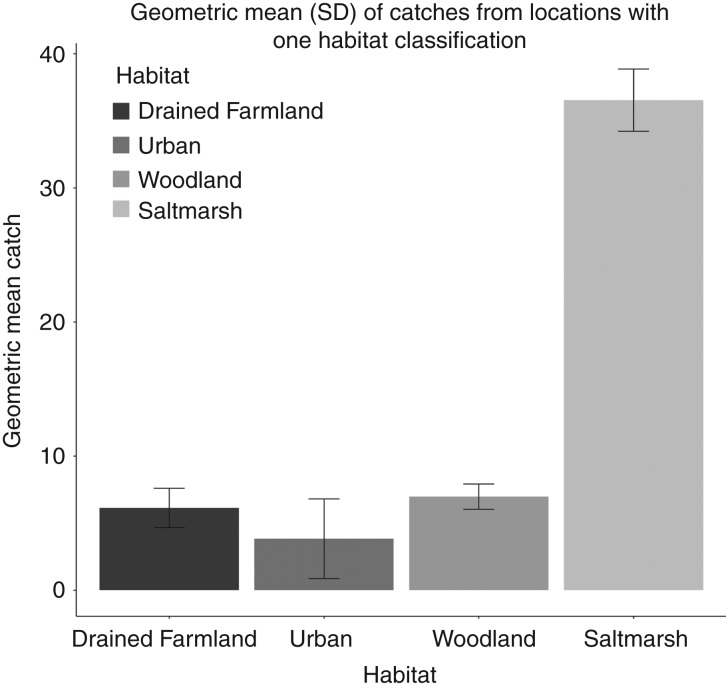
Geometric mean of total catch per location for each habitat type (locations only included if given one habitat)

**FIG 4:**
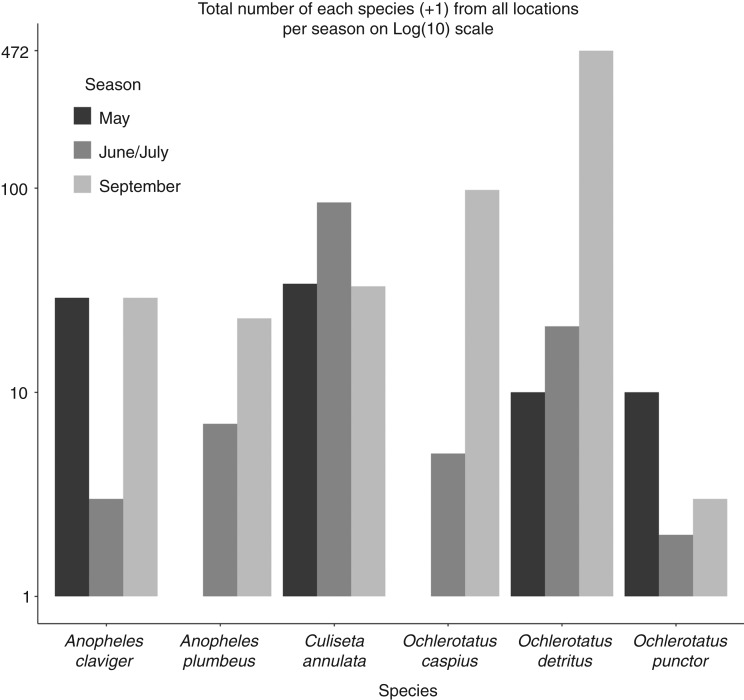
Total adult catches by season for each of six most abundant species

Due to the skewed distribution of the catch data, geometric mean is used for comparison in the text and Fig 3. Fig 4, showing total catch counts, is displayed with a log(10) scale.

## Results

### Host-seeking adults

It was not possible to find drained farmland in the southwest area sampled, so two more exposed hillside sites were chosen as a comparison (sites 18 and 19, at altitudes of 120  and 114 m, respectively). At one of these hillside locations (location 19, Table 3), trapping was not carried out in September 2015 due to loss of the propane canister. A number of specimens could not be identified positively to species level due to trap damage and are recorded as unidentified *Aedes* species.

**TABLE 3:** Adult mosquito species and number trapped in mosquito magnet trap

			Most abundantly caught mosquito species						
Location number and region	Habitats	*Anopheles claviger*	*Anopheles plumbeus*	*Culiseta annulata*	*Ochlerotatus caspius*	*Ochlerotatus detritus*	*Ochlerotatus punctor*	Other	Total
NW 1	D	0	0	12	0	0	0		12
NW 2	U	6	0	7	24	1	0	*UA—*5	43
NW 3	D	5	0	0	0	0	0		5
NW 4	U, S	0	1	12	3	53	0	*UA—*5	74
NW 5	W	0	1	5	0	2	0	*OCA*—3	11
NW 6	W	0	0	8	3	17	0		28
NW 7	S	0	0	14	1	176	0	*UA—*4	195
NW 8	W, S	3	11	12	4	85	0	*UA—*4	119
NW 9	U	0	0	1	0	0	0		1
NW 10	W, D	16	0	15	2	0	10	*OCA—*3, *CR—*2*, UA-*9 *AV—*3	60
NE 11	W	0	0	6	0	0	0		6
NE 12	W, U	1	0	20	0	0	0	*CuS—*1*, CR—*1	23
NE 13	S	8	0	2	19	0	0		29
NE 14	S	5	0	0	3	1	0	*CR—*3	12
NE 15	D	6	0	15	0	0	0	*AnM—*1*, CR—*1 *UA—*1	24
NE 16	U	3	0	2	0	0	0		5
SW 17	W	1	0	2	0	0	0	*AnM—*1	4
SW 18	H	0	0	0	0	0	0		0
SW 19	H	0	0	0	0	0	0	*CxP—*1	1
SW 20	W, S	3	0	2	0	4	0	*CxP—*1	10
SW 21	S, U	0	1	0	8	0	0	*CxP—*1*, UA—*8	18
SW 22	W, U	0	0	0	0	1	2		3
SW 23	W	0	13	1	0	0	0		14
SW 24	W	0	1	1	0	0	0	*CxP—*1	3
SE 25	U	0	0	0	0	0	0	*CxP—*1	1
SE 26	W	0	0	1	0	0	0	*OD*—1	2
SE 27	W, U	0	0	0	0	0	0	*CR—*1	1
SE 28	W	0	0	6	0	0	0	*OR*—3	9
SE 29	D	0	0	3	1	0	0	*OR*—2	6
SE 30	S	0	0	4	33	155	0		192
SE 31	S	1	0	2	0	2	0		5
SE 32	D	0	0	1	0	0	0		1

*AnM*, *An. maculipennis*; *AV*, *Ae. vexans*; *CR*, *Cq. richiardii*; *CuS*, *Cs. subochrea*; *CxP*, *Cx. pipiens s.l.*; D, drained farmland; NE, northeast; NW, northwest; *OCA*, *Oc. cantans*; *OD*, *Oc. dorsalis*; *OR*, *Oc. rusticus*; S, salt marsh; SE, southeast; SW, southwest; U, urban; *UA*, unidentified *Aedes*species; W, woodland

A total of 917 adult mosquitoes of 14 species were caught over a total of 285 trapping days over the 32 locations (Table 3). The geometric mean catch for each mosquito magnet trapping period (approximately 72 hours) was 3.7 (sd 3.4), across all locations and seasons. Totals caught were 487, 217, 160 and 53 in the areas sampled in the northwest, southeast, northeast and southwest, respectively.

For locations given one habitat classification, the geometric mean catch (nine days across three sampling periods) from a mosquito magnet was 6.9 (sd 5.90), 3.8 (2.5), 6.1 (3.3) and 36.5 (5.2) for premises associated with woodland, urban, drained farmland and saltmarsh habitats, respectively (Fig 3).

The most abundantly trapped species was *Oc. detritus* with a total of 499 adults caught. All three sites with total catches >100 were associated with the saltmarsh habitat of this species.

The second most abundantly trapped species was *Cs. annulata*, with 154 adults caught. *Cs. annulata* had the highest presence and was trapped on 75 per cent (24/32) of sites.

Total catch was highest in September (Fig 4), and the difference in catch was significantly higher (P<0.005) than that in May and that in June/July in a general linear model with a negative binomial distribution using the R statistical programme and the MASS package (The R Foundation (2016) R: The R Project for Statistical Computing. The R Foundation. https://www.r-project.org/. Accessed June 15, 2016; Ripley and others 2016). Thirty-one sites were sampled (one site not sampled), and total mosquito number from all locations was 679 with a geometric mean of 5.6 (sd 5.1) per location.

No mosquitoes were trapped while feeding on horses. Only three blood-fed mosquitoes were trapped, all were part-fed individuals caught in the mosquito magnet, of which two were *Oc. detritus* and one was *Cs. annulata.* One mosquito (*Oc. caspius*) was sampled landing on a human host. Pilot host-landing catches using horses carried out in September 2014 yielded 20 *Oc. detritus,* 3 *Oc. caspius* and 2 *Cs. annulata*, in two 15-minute daytime sampling efforts at site 8.

### Resting adults

Sampling of resting mosquitoes was unsuccessful. No mosquitoes were found in the red-box traps.

### Immature mosquitoes

Immature mosquitoes were recovered by dipping of water sources on 23 of 32 premises. A total of 61 samples containing mosquito larvae or pupae were collected from a variety of water sources including ditches, buckets and water butts, tyres, ruts, muck heaps, pools and ponds.

*Cx. pipiens s.l., Cx. torrentium, Cs. annulata/alaskaensis/subochrea, Culiseta fumipennis, Cs. morsitans, Oc. caspius, Anopheles claviger* and *An. maculipennis s.l.* were captured using dipping techniques.

The majority of samples were from artificial containers with small amounts of water, such as tyres. Therefore on most occasions, samples from each container were less than 500 ml, so it was not considered appropriate to state the numbers sampled, nor possible to compare larval numbers across sites. Larval samples were used to identify the presence of a species rather than its relative abundance.

A selection of larvae identified morphologically as *Cx. pipiens/torrentium* was further identified by molecular methods for each location. Of the 23 sites from which samples were obtained, *Cx. pipiens* larvae were identified from 65.2 per cent of locations, *Cx. torrentium* from 47.8 per cent. Both species were found on 21.7 per cent of these 23 locations. Both *Cx. pipiens* and *Cx. torrentium* larvae were obtained from at least two sites in all four regions.

*Cs. annulata/alaskaensis/subochrea* larvae cannot be differentiated morphologically and were obtained at 28.1 per cent (9/32) of sites. Due to the rarity of *Cs. alaskaensis* and the relative abundance of *Cs. annulata,* it is likely that the vast majority of these were *Cs. annulata*. The total of sites with presence of *Cs. annulata/alaskaensis/subochrea* including adult samples was 84.4 per cent (27/32).

## Discussion

This study is, to the authors’ knowledge, the first survey of mosquito species on equine premises in the UK. This work has demonstrated the presence of several mosquito species that are candidate vectors of pathogens affecting horses. Commonly found mosquito species on equine premises during this study included *Oc. detritus*, *Oc. caspius, Cs. annulata, Cx. pipiens s.l*., *Cx. torrentium, An. claviger, An. plumbeus* and *Oc. punctor.* Although mosquito density could be considered low at most of the sites sampled, this could be partly explained by the fact that the months of March, April and May in 2015 were relatively dry for all of the regions except the northwest (Met Office 2016). Climate change predictions suggest increased temperature and potential for flooding events in the UK (Met Office 2010, Caminade and others 2012, Medlock and Leach 2015), which are likely to increase the abundance of native mosquito species. It therefore seems likely that in the future there may be significantly increased horse–vector interaction, particularly with mosquito species that thrive in warmer regions of Europe, such as *Cx. annulata, Oc. caspius, Cx. pipiens s.l. Oc. detritus, An. plumbeus, Cq. richiardii, An. maculipennis and Ae. vexans* (Balenghien and others 2008)*.* The species trapped in the current study are all considered mammalophilic or bite both birds and mammals, with the exception of *Cx. torrentium* that is strongly ornithophilic (bird biting). Three European studies provide evidence that *Cx. pipiens s.l*. found in rural areas will bite mammals, including horses (Balenghien and others 2008, Börstler and others 2016, Schönenberger and others 2016). Although not all of these studies differentiated *Cx. pipiens* form *pipiens* from *Cx. pipiens* form *molestus,* the study of Börstler and others (2016) records a significant number of *Cx. pipiens* form *pipiens* with mammalian blood meals. *Oc. detritus*, *Oc. caspius, Cx. pipiens s.l*., *Oc. punctor* and *An. plumbeus* have all been shown to transmit arboviruses affecting horses and therefore are important when considering the risk of mosquito-borne equine disease. Also, 11 of the 16 species found on equine premises during this study are laboratory-competent vectors of, or are implicated in, naturally occurring disease cycles for at least one arbovirus-affecting horses (Table 2). An important aspect of this study is that the authors trapped very few blood-fed mosquitoes: just three in the mosquito magnet and none by other methods. This begs the question of whether the mosquitoes present at equine premises in the UK only rarely feed on equines or whether they feed but were not caught. A number of factors suggest that the latter is the most likely explanation: (i) the mosquito magnet is designed to trap host-seeking rather than blood-fed adults; (ii) many of the premises had other potential hosts present (human beings, cattle, small mammals), indicating that the low number of trapped blood-fed mosquitoes cannot be attributed to the specific avoidance of equids; (iii) in pilot work in September 2014, mosquitoes *Cs. annulata, Oc. caspius and Oc. detritus* were directly observed by the author feeding on horses; and (iv) most of the species caught in this study have been reported, in other studies, to feed on horses and/or transmit arboviruses to horses. Nevertheless, and probably due to the inherent difficulties in trapping blood-fed mosquitoes in the UK (Brugman and others 2015), blood-feeding on horses has not been confirmed in this study. A large sampling effort and high mosquito densities are required to maximise trapping of blood-fed mosquitoes. The number of sites included in this study dictated that sampling effort on each site was necessarily lower than that of other recent studies (Brugman 2016), and seasonal variation in abundance due to climatic conditions, for example, a dry early summer period (Met Office 2016), may have supressed mosquito density. However, all of the species sampled in this study, with the exception of *Cx. torrentium* and *Cs. morsitans,* have been shown to bite equines (Table 2), and four of the six most abundant species in adult catches have been shown to bite horses in the UK either in previous studies or in pilot work for this study. Further work would be required to investigate the feeding rate of UK populations of these mosquitoes on horses, and host bait catches (Schönenberger and others 2016) would seem most likely to provide useful information.

The comparatively high numbers of *Oc. detritus* and *Oc. caspius* caught on some saltmarsh-associated sites are consistent with previous studies and reports of significant nuisance biting (Clarkson and Setzkorn 2011, Medlock and others 2012, Medlock and Vaux 2013) and confirm that there is significant potential for host–vector interaction between these species and horses. These two species are competent vectors of WNV (Vermeil and others 1960, Blagrove and others, 2016). Detailed, high resolution information regarding horse and mosquito species distribution is lacking (Lo Iacono and others 2013). However, using previously published horse distribution data at postcode scale (Boden and others 2012, Lo Iacono and others 2013) and saltmarsh distribution (Adnitt and others 2007), in combination with mosquito species records, several coastal areas of England appear worthy of further investigation for host–vector interaction potential. These areas have high horse density, saltmarsh presence and records of *Oc. detritus* and *Oc. caspius* (The Walter Reed Biosystematics Unit 2014, National Biodiversity Network 2016a, b) and include the Severn estuary, South Devon coast, the south coast of England from Swanage to Chichester and the Dee and Mersey estuaries. Two of these areas were sampled during this study: Wirral (Dee estuary) and the South Devon coast.

The finding that the WNV vector *Cx. pipiens* was common on equine premises with suitable water sources is expected as this species has a widespread distribution (Medlock and others 2005, Medlock and Vaux 2011), but this study confirms that suitable container habitats are commonplace on equine premises. *Cx. torrentium* is a major enzootic (wildlife) vector of Sindbis virus in Scandinavia (Hesson and others 2015) and may therefore be capable of a similar role in transmission of other arboviruses. *Cx. pipiens* and *Cx. torrentium* were found on a number of occasions in all four regions, suggesting that *Cx. torrentium* may be more prevalent in the north of England than previously recognised (Medlock and others 2005).

One of the most interesting results to emerge from the current study was the presence of *Cs. annulata* on the majority of sites (27/32). It was also the second must abundant species in mosquito magnet samples, after *Oc. detritus*. While *Cs. annulata* is known to have a widespread distribution in the UK (Medlock and others 2005), this study provides evidence of the potential for host–vector interaction with UK equines. *Cs. annulata* has recently been demonstrated to be vector competent for WNV (M. S. C. Blagrove, personal communication), and as the species bites both birds and mammals including horses (Schönenberger and others 2016), it therefore has potential to transmit arboviruses from avian reservoirs and hence serve as a ‘bridge vector’. Combined with its ability to breed in a variety of water sources and presence on most sites sampled, this makes it an important species for further study.

The authors’ results suggest that mosquito species presence is determined mainly by local mosquito breeding habitat rather than equine host availability or management factors. However, biting of horses may be affected by practices such as use of repellents, rugs and masks, building design, and duration and timing of grazing.

Mosquito magnets are a commonly used trap in Europe for surveillance. They catch almost all mammalophagic species of mosquito, and catch more species than other systems and in greater numbers. BG sentinel and Centers for Disease Control and Prevention traps were not used in this study as they were considered less suitable due to the risk of unpredictable precipitation damaging samples, and because for wide-scale trapping in the UK, it may prove more practicable to use propane vendor's delivery services than to transport large amounts of dry ice or carbon dioxide. Red-box traps were used in the current study to attempt to trap blood-fed mosquitoes; however, no mosquitoes were captured. Similar but larger red-box traps have been successful in capturing *An. maculipennis s.l*., *Cs. annulata* and *Culex* species in England (Brugman 2016). At similar future levels of mosquito abundance and without the presence of invasive species, surveillance on equine premises in the UK should be based around the use of mosquito magnets and larval sampling.

Mosquito populations often have a patchy distribution (Medlock and others 2005, Snow and Medlock 2008, Golding 2013) and many are considered uncommon or rare. Simple random sampling of equine premises may have resulted in very low catches. It is also almost impossible to prove species absence, so using random sampling risked obtaining poor quality data. Stratified sampling is an alternative method, commonly used by ecologists studying rare species (Thompson 2012). Using the data obtained under this sampling regimen, it is not possible to estimate the risk of equine–mosquito interaction across the UK, but more accurate assessment of risk at individual sites based on local habitat is achievable.

There are a number of introduction pathways that could conceivably be involved in importation of arboviral disease to the UK. Perhaps the most often discussed is introduction of WNV by migratory birds, but trade and transport of exotic birds and pets, and inadvertent vector transportation are also relevant risks. There is some recent evidence that human populations may continue epidemic transmission of VEEV in urban environments (Bowen and Calisher 1976, Watts and others 1998, Morrison and others 2008). Therefore in the event of an outbreak in the Americas, human movements as well as horse movements may constitute a risk (Adams and others 2012). Livestock transport, human transport and possibly mosquito eggs may present risk of RRV introduction (Harley and others 2001). Due to the complexity of the transmission cycles, virus introduction may not result in autochthonous (in-country) transmission.

In conclusion, the current study has highlighted a number of mosquito species that should be investigated with regard to vector competence and effectiveness of protection measures for equines. The authors’ work has shown that horses in the UK are at risk of attack from a wide variety of mosquito species, several of which are known to be vectors of equine arboviruses in affected countries.

## References

[R1] ADAMS A. P., NAVARRO-LOPEZ R., RAMIREZ-AGUILAR F. J., LOPEZ-GONZALEZ I., LEAL G., FLORES-MAYORGA J. M., TRAVASSOS DA ROSA A. P. A., SAXTON-SHAW K. D., SINGH A. J., BORLAND E. M., POWERS A. M., TESH R. B., WEAVER S. C. & ESTRADA-FRANCO J. G. (2012) Venezuelan Equine Encephalitis Virus Activity in the Gulf Coast Region of Mexico, 2003–2010. PLoS Neglected Tropical Diseases 6, e1875 10.1371/journal.pntd.0001875 23133685 PMC3486887

[R2] ADNITT C., GREAT BRITAIN, ENVIRONMENT AGENCY and DEFRA/ENVIRONMENT AGENCY FLOOD AND COASTAL EROSION RISK MANAGEMENT R & D PROGRAMME (GREAT BRITAIN) (2007) Saltmarsh Management Manual. Environment Agency

[R3] ANDREADIS T. G., ANDERSON J. F. & TIRRELL-PECK S. J. (1998) Multiple isolations of eastern equine encephalitis and highlands J viruses from mosquitoes (Diptera: Culicidae) during a 1996 epizootic in southeastern Connecticut. Journal of Medical Entomology 35, 296–302 10.1093/jmedent/35.3.296 9615549

[R4] ARMSTRONG P. M. & ANDREADIS T. G. (2010) Eastern equine encephalitis virus in mosquitoes and their role as bridge vectors. Emerging Infectious Diseases 16, 1869–1874 21122215 10.3201/eid1612.100640 PMC3294553

[R6] AVILES G., SABATTINI M. S. & MITCHELL C. J. (1990) Peroral susceptibility of Aedes albifasciatus and Culex pipiens complex mosquitoes (Diptera: Culicidae) from Argentina to western equine encephalitis virus. Revista de Saúde Pública 24, 265–269 10.1590/S0034-89101990000400003 2103643

[R7] BALENGHIEN T., SABATIER P., BICOUT D. J. & FOUQUE F. (2006) Horse-, bird-, and human-seeking behavior and seasonal abundance of mosquitoes in a West Nile virus focus of Southern France. Journal of Medical Entomology 43, 936–946 10.1093/jmedent/43.5.936 17017231

[R8] BALENGHIEN T., VAZEILLE M., GRANDADAM M., SCHAFFNER F., ZELLER H., REITER P., SABATIER P., FOUQUE F. & BICOUT D. J. (2008) Vector Competence of Some French *Culex* and *Aedes* Mosquitoes for West Nile Virus. Vector-Borne and Zoonotic Diseases 8, 589–596 10.1089/vbz.2007.0266 18447623

[R9] BECKER N., PETRIĆ D., BOASE C., LANE J., ZGOMBA M., DAHL C. & KAISER A. (2010) Mosquitoes and Their Control. 2nd edn Springer http://link.springer.com/content/pdf/10.1007/978-3-540-92874-4.pdf. Accessed September 20, 2014

[R10] BODEN L. A., PARKIN T. D., YATES J., MELLOR D. & KAO R. R. (2012) Summary of current knowledge of the size and spatial distribution of the horse population within Great Britain. BMC Veterinary Research 8, 43 10.1186/1746-6148-8-43 22475060 PMC3351363

[R11] BÖRSTLER J., JÖST H., GARMS R., KRÜGER A., TANNICH E., BECKER N., SCHMIDT-CHANASIT J. & LÜHKEN R. (2016) Host-feeding patterns of mosquito species in Germany. Parasites and Vectors 9, 318 10.1186/s13071-016-1597-z 27259984 PMC4893232

[R12] BOWEN G. S. & CALISHER C. H. (1976) Virological and serological studies of Venezuelan equine encephalomyelitis in humans. Journal of Clinical Microbiology 4, 22–27 956360 10.1128/jcm.4.1.22-27.1976 PMC274383

[R13] BROWN H. E., PALADINI M., COOK R. A., KLINE D., BARNARD D. & FISH D. (2008) Effectiveness of mosquito traps in measuring species abundance and composition. Journal of Medical Entomology 45, 517–521 doi:10.1603/0022-2585(2008)45[517:EOMTIM]2.0.CO;2 18533447 10.1603/0022-2585(2008)45[517:eomtim]2.0.co;2

[R14] BRUGMAN V. A. (2016) Host selection and feeding preferences of farm-associated mosquitoes (Diptera: Culicidae) in the United Kingdom. London, UK: University of London.

[R15] BRUGMAN V. A., HERNÁNDEZ-TRIANA L. M., PROSSER S. W. J., WELAND C., WESTCOTT D. G., FOOKS A. R. & JOHNSON N. (2015) Molecular species identification, host preference and detection of myxoma virus in the Anopheles maculipennis complex (Diptera: Culicidae) in southern England, UK. Parasites & Vectors 8, 421 10.1186/s13071-015-1034-8 26271277 PMC4536751

[R16] CAMINADE C., MEDLOCK J. M., DUCHEYNE E., MCINTYRE K. M., LEACH S., BAYLIS M. & MORSE A. P. (2012) Suitability of European climate for the Asian tiger mosquito Aedes albopictus: recent trends and future scenarios. Journal of The Royal Society Interface 9, 2708–2717 10.1098/rsif.2012.0138 PMC3427500 22535696

[R17] CENTERS FOR DISEASE CONTROL AND PREVENTION (CDC) (2006) Eastern equine encephalitis—New Hampshire and Massachusetts, August–September 2005. MMWR. Morbidity and Mortality Weekly Report 55, 697–700 16810146

[R18] CHAMBERLAIN R. W., BIKES R. K., NELSON D. B. & SUDIA W. D. (1954) Studies on the North American Arthropod-Borne Encephalitides VI. Quantitative Determinations of Virus–Vector Relationships. American Journal of Epidemiology 60, 278–285 10.1093/oxfordjournals.aje.a119721 13207099

[R19] CLARKSON M. J. & SETZKORN C. (2011) The domestic mosquitoes of the Neston area of Cheshire, UK. European Mosquito Bulletin 29, 122–128

[R20] CRANSTON P. S., RAMSDALE C. D., SNOW K. R. & WHITE G. B. (1987) Keys to the Adults, Male Hypopygia, Fourth-Instar Larvae and Pupae of the British Mosquitoes (Culicidae) with Notes on their Ecology and Medical Importance. Freshwater Biological Association http://www.cabdirect.org/abstracts/19880592401.html. Accessed January 14, 2016

[R21] DANABALAN R. (2010) Mosquitoes of Southern England and Northern. Wales: Identification, Ecology and Host selection

[R22] DAVIS W. A. (1940) A Study of Birds and Mosquitoes as Hosts for the Virus of Eastern Equine Encephalomyelitis. American Journal of Hygiene 32, 45–59

[R23] DURAND B., LECOLLINET S., BECK C., MARTÍNEZ-LÓPEZ B., BALENGHIEN T. & CHEVALIER V. (2013) Identification of Hotspots in the European Union for the Introduction of Four Zoonotic Arboviroses by Live Animal Trade. PLoS ONE 8, e70000 10.1371/journal.pone.0070000 23894573 PMC3720944

[R24] ELBERS A. R. W., KOENRAADT C. J. & MEISWINKEL R. (2015) Mosquitoes and Culicoides biting midges: Vector range and the influence of climate change. OIE Revue Scientifique et Technique 34, 123–137 10.20506/rst.34.1.2349 26470453

[R25] ELLIS P. M., DANIELS P. W. & BANKS D. J. (2000) Japanese encephalitis. The Veterinary Clinics of North America. Equine Practice 16, 565–578 11219350 10.1016/s0749-0739(17)30096-2

[R26] FARAJ C., ADLAOUI E., OUAHABI S., RHAJAOUI M., FONTENILLE D. & LYAGOUBI M. (2009) Entomological investigations in the region of the last malaria focus in Morocco. Acta Tropica 109, 70–73 10.1016/j.actatropica.2008.09.021 18992211

[R27] GOLDING N. (2013) Mapping and understanding the distributions of potential vector mosquitoes in the UK: New methods and applications. Oxford, UK: University of Oxford.

[R28] GUIS H., BAYLIS M., TRAN A., CAMINADE C., MORSE A. P. & CALVETE C. (2012) Modelling the effects of past and future climate on the risk of bluetongue emergence in Europe. Journal of the Royal Society Interface 9, 339–350 10.1098/rsif.2011.0255 PMC3243388 21697167

[R29] HALE J. H. & WITHERINGTON D. H. (1953) Encephalitis in racehorses in Malaya. Journal of Comparative Pathology and Therapeutics 63, 195–198 10.1016/S0368-1742(53)80023-8 13084794

[R30] HAMMON W. M. & REEVES W. C. (1943) Laboratory transmission of the Western equine encephalomyelitis virus by mosquitoes of the gener Culex and Culiseta. The Journal of Experimental Medicine 78, 425–434 10.1084/jem.78.6.425 19871339 PMC2135424

[R31] HARLEY D., SLEIGH A. & RITCHIE S. (2001) Ross River virus transmission, infection, and disease: a cross-disciplinary review. Clinical Microbiology Reviews 14, 909–932 10.1128/CMR.14.4.909-932.2001 11585790 PMC89008

[R32] HESSON J. C., LUNDSTRÖM J. O., HALVARSSON P., ERIXON P. & COLLADO A. (2010) A sensitive and reliable restriction enzyme assay to distinguish between the mosquitoes Culex torrentium and Culex pipiens. Medical and Veterinary Entomology 24, 142–149 10.1111/j.1365-2915.2010.00871.x 20444079

[R33] HESSON J. C., VERNER-CARLSSON J., LARSSON A., AHMED R., LUNDKVIST Å. & LUNDSTRÖM J. O. (2015) *Culex torrentium* Mosquito Role as Major Enzootic Vector Defined by Rate of Sindbis Virus Infection, Sweden, 2009. Emerging Infectious Diseases 21, 875–878 10.3201/eid2105.141577 25898013 PMC4412225

[R34] HOLMES J., GILKERSON J., EL HAGE C., SLOCOMBE R. & MUURLINK M. (2012) Murray Valley encephalomyelitis in a horse. Australian Veterinary Journal 90, 252–254 10.1111/j.1751-0813.2012.00949.x 22731944

[R35] HUTCHINSON R. A. (2004) Mosquito borne diseases in England: past, present and future risks, with special reference to malaria in the Kent Marshes. http://etheses.dur.ac.uk/3067/. Accessed March 2, 2015

[R36] HUTCHINSON R. A., WEST P. A. & LINDSAY S. W. (2007) Suitability of two carbon dioxide-baited traps for mosquito surveillance in the United Kingdom. Bulletin of Entomological Research 97, 591–597 10.1017/S0007485307005263 17997872

[R37] KILPATRICK A. M. & RANDOLPH S. E. (2012) Drivers, dynamics, and control of emerging vector-borne zoonotic diseases. The Lancet 380, 1946–1955 10.1016/S0140-6736(12)61151-9 PMC3739480 23200503

[R38] KRAMER L. D., REISEN W. K. & CHILES R. E. (1998) Vector competence of Aedes dorsalis (Diptera: Culicidae) from Morro Bay, California, for western equine encephalomyelitis virus. Journal of Medical Entomology 35, 1020–1024 10.1093/jmedent/35.6.1020 9835696

[R39] LO IACONO G., ROBIN C. A., NEWTON J. R., GUBBINS S. & WOOD J. L. (2013) Where are the horses? With the sheep or cows? Uncertain host location, vector-feeding preferences and the risk of African horse sickness transmission in Great Britain. Journal of The Royal Society Interface 10, 20130194 10.1098/rsif.2013.0194 PMC3645429 23594817

[R40] LONG M. T. & GIBBS E. P. J. (2007) Chapter 20—Equine Alphaviruses. In Equine Infectious Diseases. Eds D. C. Sellon & M. T. Long 1st edn Saunders, Elsevier pp 191–197

[R41] MACKENZIE-IMPOINVIL L., IMPOINVIL D. E., GALBRAITH S. E., DILLON R. J., RANSON H., JOHNSON N., FOOKS A. R., SOLOMON T. & BAYLIS M. (2015) Evaluation of a temperate climate mosquito, *Ochlerotatus detritus* (*Aedes detritus*), as a potential vector of Japanese encephalitis virus. Medical and Veterinary Entomology. 29, 1–9 10.1111/mve.12083 25087926

[R42] MARSHALL J. F. (1938) The British Mosquitoes. London: British Museum, 1938

[R44] MEDLOCK J. M., HANSFORD K. M., ANDERSON M., MAYHO R. & SNOW K. R. (2012) Mosquito nuisance and control in the UK—A questionnaire-based survey of local authorities. European Mosquito Bulletin 30, 15–29

[R45] MEDLOCK J. M. & LEACH S. A. (2015) Effect of climate change on vector-borne disease risk in the UK. The Lancet Infectious Diseases 15, 721–730 10.1016/S1473-3099(15)70091-5 25808458

[R46] MEDLOCK J. M., SNOW K. R. & LEACH S. (2005) Potential transmission of West Nile virus in the British Isles: an ecological review of candidate mosquito bridge vectors. Medical and Veterinary Entomology 19, 2–21 10.1111/j.0269-283X.2005.00547.x 15752172

[R47] MEDLOCK J. M., SNOW K. R. & LEACH S. (2007) Possible ecology and epidemiology of medically important mosquito-borne arboviruses in Great Britain. Epidemiology and Infection 135, 466–482 10.1017/S0950268806007047 16893487 PMC2870593

[R48] MEDLOCK J. M. & VAUX A. G. (2011) Assessing the possible implications of wetland expansion and management on mosquitoes in Britain. Eur Mosq Bull 29, 38–65

[R49] MEDLOCK J. M. & VAUX A. G. C. (2013) Colonization of UK coastal realignment sites by mosquitoes: implications for design, management, and public health. Journal of Vector Ecology 38, 53–62 10.1111/j.1948-7134.2013.12008.x 23701607

[R50] MEDLOCK J. M. & VAUX A. G. C. (2014) Colonization of a newly constructed urban wetland by mosquitoes in England: implications for nuisance and vector species. Journal of Vector Ecology 39, 249–260 10.1111/jvec.12099 25424253

[R51] MEDLOCK J. M. & VAUX A. G. C. (2015) Seasonal dynamics and habitat specificity of mosquitoes in an English wetland: implications for UK wetland management and restoration. Journal of Vector Ecology 40, 90–106 10.1111/jvec.12137 26047189

[R52] MERRILL M. H., LACAILLADE C. W. & BROECK C. T. (1934) Mosquito Transmission of Equine Encephalomyelitis. American Association for the Advancement of Science http://arch.neicon.ru/xmlui/handle/123456789/2596223. Accessed February 2, 2015 10.1126/science.80.2072.251 17800857

[R53] MET OFFICE (2010) Met Office UKCP. http://ukclimateprojections.metoffice.gov.uk/. Accessed June 20, 2016

[R54] MET OFFICE (2016) UK actual and anomaly maps. http://www.metoffice.gov.uk/climate/uk/summaries/anomacts. Accessed June 20, 2016

[R55] MORRIS C. D. (1981) A structural and operational analysis of diurnal resting shelters for mosquitoes (Diptera: Culicidae). Journal of Medical Entomology 18, 419–424 10.1093/jmedent/18.5.419

[R56] MORRISON A. C., FORSHEY B. M., NOTYCE D., ASTETE H., LOPEZ V., ROCHA C., CARRION R., CAREY C., EZA D., MONTGOMERY J. M. & KOCHEL T. J. (2008) Venezuelan Equine Encephalitis Virus in Iquitos, Peru: Urban Transmission of a Sylvatic Strain. PLoS Neglected Tropical Diseases 2, e349 10.1371/journal.pntd.0000349 19079600 PMC2593782

[R57] NAKAMURA H. (1972) Japanese encephalitis in horses in Japan. Equine Veterinary Journal 4, 155–156 10.1111/j.2042-3306.1972.tb03900.x 4346982

[R58] NATIONAL BIODIVERSITY NETWORK (2016a) NBN Grid Map for Aedes caspius (Pallas, 1771). https://data.nbn.org.uk/Taxa/NBNSYS0000011577/Grid_Map. Accessed March 17, 2016

[R59] NATIONAL BIODIVERSITY NETWORK (2016b) NBN Grid Map for Aedes detritus (Halliday, 1883). https://data.nbn.org.uk/Taxa/NBNSYS0000011579/Grid_Map. Accessed March 17, 2016

[R60] PAGES N., HUBER K., CIPRIANI M., CHEVALLIER V., CONRATHS F., GOFFREDO M. & BALENGHIEN T. (2009) SCIENTIFIC REPORT submitted to EFSA: Scientific review on mosquitoes and mosquito-borne diseases. *EFSA Supporting Publication* **6**, EN-7, 1–96.

[R61] PAUVOLID-CORRÊA A., TAVARES F. N., COSTA E. V., BURLANDY F. M., MURTA M., PELLEGRIN A. O., NOGUEIRA M. F. & SILVA E. E. (2010) Serologic evidence of the recent circulation of Saint Louis encephalitis virus and high prevalence of equine encephalitis viruses in horses in the Nhecolândia sub-region in South Pantanal, Central-West Brazil. Memorias do Instituto Oswaldo Cruz 105, 829–833 10.1590/S0074-02762010000600017 20945001

[R62] REMPEL J. G., RIDDELL W. A. & MCNELLY E. M. (1946) Multiple feeding habits of Saskatchewan mosquitoes. Canadian Journal of Research 24, 71–78 10.1139/cjr46e-009 21023963

[R63] RICO-HESSE R. (2000) Venezuelan equine encephalomyelitis. The Veterinary clinics of North America. Equine Practice 16, 553–563 11219349 10.1016/s0749-0739(17)30095-0

[R64] RIPLEY, B., VENABLES, B., BATES, D. M. (1998), HORNIC, K. (partial port ca, 1998), GEBHARDT, A. (partial port ca 1998) & FIRTH, D. (2016) MASS: Support Functions and Datasets for Venables and Ripley's MASS. https://cran.r-project.org/web/packages/MASS/index.html. Accessed June 15, 2016

[R65] SCHAFFNER E., ANGEL G., GEOFFROY B., HERVY J. P., RHAIEM A. & BRUNHES J. (2001) Les moustiques d'Europe: logiciel d'identification et d'enseignement. The mosquitoes of Europe: an identification and training programme. http://www.documentation.ird.fr/hor/fdi:010027372. Accessed January 14, 2016

[R66] SCHÖNENBERGER A. C., WAGNER S., TUTEN H. C., SCHAFFNER F., TORGERSON P., FURRER S., MATHIS A. & SILAGHI C. (2016) Host preferences in host-seeking and blood-fed mosquitoes in Switzerland. Medical and Veterinary Entomology 30, 39–52 10.1111/mve.12155 26685926

[R67] SELLON D. C. & LONG M. (2013) Equine Infectious Diseases. Elsevier Health Sciences

[R68] SERVICE M. W. (1969) Observations on the ecology of some British mosquitoes. Bulletin of Entomological Research 59, 161–194 10.1017/S000748530000314X

[R69] SERVICE M. W. (1971a) Feeding behaviour and host preferences of British mosquitoes. Bulletin of Entomological Research 60, 653–661 10.1017/S0007485300042401 22894871

[R70] SERVICE M. W. (1971b) Flight periodicities and vertical distribution of Aedes cantans (Mg.), Ae. geniculatus (OI.), Anopheles piumbeus Steph. and Culex pipiens L. (Dipt., Culicidae) in southern England. Bulletin of Entomological Research 60, 639–651 10.1017/S0007485300042395 22894870

[R71] SERVICE M. W. (1977) Ecological and Biological Studies on Aedes cantans (Meig.) (Diptera: Culicidae) in Southern England. The Journal of Applied Ecology 14, 159–196 10.2307/2401833

[R72] SERVICE M. W. & SMITH G. (1972) Notes on the biology of Aedes Flavescens (Muller) (Dipt., Culicidae) in England. Entomologist's Monthly Magazine 108, 35–37

[R73] SERVICE M. W., VOLLER A. & BIDWELL D. E. (1986) The enzyme-linked immunosorbent assay (ELISA) test for the identification of blood-meals of haematophagous insects. Bulletin of Entomological Research 76, 321–330 10.1017/S0007485300014796

[R74] SILVA M. L. C. R., GALIZA G. J. N., DANTAS A. F. M., OLIVEIRA R. N., IAMAMOTO K., ACHKAR S. M. & RIET-CORREA F. (2011) Outbreaks of Eastern equine encephalitis in northeastern Brazil. Journal of Veterinary Diagnostic Investigation 23, 570–575 10.1177/1040638711403414 21908293

[R75] SNOW K. & MEDLOCK J. M. (2008) The mosquitoes of Epping Forest, Essex, UK. European Mosquito Bulletin 26, 9–17

[R76] SNOW K. R. (1991) Mosquitoes. Richmond, Surrey: Richmond Publishing Co

[R77] SPICKLER A. R. (2010) Emerging and Exotic Diseases of Animals. CFSPH Iowa State University https://books.google.co.uk/books?hl=en&lr=&id=iiAqA4AD6zsC&oi=fnd&pg=PP1&dq=Emerging+and+exotic+diseases+of+animals&ots=caiVnmCvvN&sig=2oc-66l0_IAZF6BLq9bp9V9NV-E. Accessed August 3, 2015

[R78] SUDIA W. D., MCLEAN R. G., NEWHOUSE R. G., JOHNSTON J. G., MILLER D. L., TREVINO H., BOWEN G. S. & SATHER G. (1975) Epidemic Venezuelan Equine Encephalitis in North America in 1971: Vertebrate Field Studies. American Journal of Epidemiology 101, 36–50 1119481 10.1093/oxfordjournals.aje.a112069

[R79] THE WALTER REED BIOSYSTEMATICS UNIT (2014) VectorMap Data Portal. http://vectormap.si.edu/dataportal.htm. Accessed March 21, 2016

[R80] THOMPSON S. K. (2012) Chapter 11. Stratified sampling. In Sampling. 3rd edn Wiley pp 141–156

[R81] TURELL M. J. (2012) Members of the Culex pipiens Complex as Vectors of Viruses. Journal of the American Mosquito Control Association 28, 123–126 10.2987/8756-971X-28.4.123 23401952

[R82] TURELL M. J., MORES C. N., DOHM D. J., KOMILOV N., PARAGAS J., LEE J. S., SHERMUHEMEDOVA D., ENDY T. P., KODIROV A. & KHODJAEV S. (2006) Laboratory transmission of Japanese encephalitis and West Nile viruses by molestus form of Culex pipiens (Diptera: Culicidae) collected in Uzbekistan in 2004. Journal of Medical Entomology 43, 296–300 doi:10.1603/0022-2585(2006)043[0296:LTOJEA]2.0.CO;2 16619614 10.1603/0022-2585(2006)043[0296:ltojea]2.0.co;2

[R83] VAIDYANATHAN R., EDMAN J. D., COOPER L. A. & SCOTT T. W. (1997) Vector competence of mosquitoes (Diptera: Culicidae) from Massachusetts for a sympatric isolate of eastern equine encephalomyelitis virus. Journal of Medical Entomology 34, 346–352 10.1093/jmedent/34.3.346 9151501

[R84] VALE T., SPRATT D. & CLOONAN M. (1991) Serological Evidence of Arbovirus Infection in Native and Domesticated Mammals on the South Coast of New-South-Wales. Australian Journal of Zoology 39, 1–7 10.1071/ZO9910001

[R85] VARDOULAKIS S. & HEAVISIDE C. (2012) Health Effects of Climate Change in the UK 2012. Health Protection Agency http://www.climatenorthernireland.org.uk/cmsfiles/resources/files/Health-Effects-of-Climate-Change-in-the-UK-2012_Department-of-Health.pdf. Accessed February 20, 2016

[R86] VAUX A. G., GIBSON G., HERNANDEZ-TRIANA L. M., CHEKE R. A., MCCRACKEN F., JEFFRIES C. L., HORTON D. L., SPRINGATE S., JOHNSON N., FOOKS A. R., LEACH S. & MEDLOCK J. M. (2015) Enhanced West Nile virus surveillance in the North Kent marshes, UK. Parasites & Vectors 8, 91 10.1186/s13071-015-0705-9 25884920 PMC4342892

[R87] VAUX A. G. C. & MEDLOCK J. M. (2015) Current status of invasive mosquito surveillance in the UK. Parasites & Vectors 8, 351 10.1186/s13071-015-0936-9 26122427 PMC4491199

[R88] VERDONSCHOT P. F. M. & BESSE-LOTOTSKAYA A. A. (2014) Flight distance of mosquitoes (Culicidae): a metadata analysis to support the management of barrier zones around rewetted and newly constructed wetlands. Limnologica-Ecology and Management of Inland Waters 45, 69–79 10.1016/j.limno.2013.11.002

[R89] VERMEIL C., LAVILLAUREIX J. & BEEB E. (1960) Sur la conservation et la transmission du virus West Nile par quelques arthropodes. Bulletin de la Societe de Pathologie Exotique 53, 273–279 13780928

[R90] WATTS D. M., CALLAHAN J., ROSSI C., OBERSTE M. S., ROEHRIG J. T., WOOSTER M. T., SMITH J. F., CROPP C. B., GENTRAU E. M., KARABATSOS N., GÜBLER D. & HAYES C. G. (1998) Venezuelan equine encephalitis febrile cases among humans in the Peruvian Amazon River region. The American Journal of Tropical Medicine and Hygiene 58, 35–40 9452289 10.4269/ajtmh.1998.58.35

[R91] ZACKS M. A. & PAESSLER S. (2010) Encephalitic alphaviruses. Veterinary Microbiology 140, 281–286 10.1016/j.vetmic.2009.08.023 19775836 PMC2814892

[R92] ZEHMER R. B., DEAN P. B., SUDIA W. D., CALISHER C. H., SATHER G. E. & PARKER R. L. (1974) Venezuelan equine encephalitis epidemic in Texas, 1971. Health Services Reports 89, 278 10.2307/4595031 4151413 PMC1616214

